# L-Arginine Destabilizes Oral Multi-Species Biofilm Communities Developed in Human Saliva

**DOI:** 10.1371/journal.pone.0121835

**Published:** 2015-05-06

**Authors:** Ethan Kolderman, Deepti Bettampadi, Derek Samarian, Scot E. Dowd, Betsy Foxman, Nicholas S. Jakubovics, Alexander H. Rickard

**Affiliations:** 1 Center for Molecular and Clinical Epidemiology of Infectious Diseases, Department of Epidemiology, University of Michigan, Ann Arbor, MI, United States of America; 2 Molecular Research LP (MR DNA), Shallowater, TX, United States of America; 3 Centre for Oral Health Research, School of Dental Sciences, Newcastle University, Newcastle upon Tyne, NE2 4BW, United States of America; University Hospital of the Albert-Ludwigs-University Freiburg, GERMANY

## Abstract

The amino acid L-arginine inhibits bacterial coaggregation, is involved in cell-cell signaling, and alters bacterial metabolism in a broad range of species present in the human oral cavity. Given the range of effects of L-arginine on bacteria, we hypothesized that L-arginine might alter multi-species oral biofilm development and cause developed multi-species biofilms to disassemble. Because of these potential biofilm-destabilizing effects, we also hypothesized that L-arginine might enhance the efficacy of antimicrobials that normally cannot rapidly penetrate biofilms. A static microplate biofilm system and a controlled-flow microfluidic system were used to develop multi-species oral biofilms derived from pooled unfiltered cell-containing saliva (CCS) in pooled filter-sterilized cell-free saliva (CFS) at 37^o^C. The addition of pH neutral L-arginine monohydrochloride (LAHCl) to CFS was found to exert negligible antimicrobial effects but significantly altered biofilm architecture in a concentration-dependent manner. Under controlled flow, the biovolume of biofilms (μm^3^/μm^2^) developed in saliva containing 100-500 mM LAHCl were up to two orders of magnitude less than when developed without LAHCI. Culture-independent community analysis demonstrated that 500 mM LAHCl substantially altered biofilm species composition: the proportion of *Streptococcus* and *Veillonella* species increased and the proportion of Gram-negative bacteria such as *Neisseria* and *Aggregatibacter* species was reduced. Adding LAHCl to pre-formed biofilms also reduced biovolume, presumably by altering cell-cell interactions and causing cell detachment. Furthermore, supplementing 0.01% cetylpyridinium chloride (CPC), an antimicrobial commonly used for the treatment of dental plaque, with 500 mM LAHCl resulted in greater penetration of CPC into the biofilms and significantly greater killing compared to a non-supplemented 0.01% CPC solution. Collectively, this work demonstrates that LAHCl moderates multi-species oral biofilm development and community composition and enhances the activity of CPC. The incorporation of LAHCl into oral healthcare products may be useful for enhanced biofilm control.

## Introduction

Dental plaque biofilms are surface-associated microbial communities that are bathed in flowing saliva and typically contain tens to hundreds of bacterial species [1]. Oral biofilm architecture, species composition, and spatial arrangement of the contained species impact growth-rates and can enhance tolerance to adverse environmental conditions [2,3,4]. Depending upon the location (supragingival versus subgingival), the biomass (number of bacteria), the species composition (types and relative abundance), and spatial arrangement of the constituent species (in three dimensions), dental plaque biofilms can cause caries or periodontal disease [2,5,6]. Dental plaque biofilm communities are extremely recalcitrant to external chemical and physical perturbations. For example, they are up to 1,000 times less susceptible to antimicrobials compared to their planktonic counterparts, and are typically resistant to abrasive treatments [7,8]. Difficulties in treating oral biofilm communities, especially those causing dental caries and periodontal disease, imparts a considerable health burden in the US: approximately 500 million visits to dental offices and an estimated cost of $108 billion per annum to treat or prevent oral disease [9].

In recent years, there have been concerns with the possible overuse of antibiotics and biocides to maintain or improve oral health [10,11,12]. Adaptation of bacteria to these antimicrobials and the spread of genetic resistances via the horizontal exchange of antimicrobial resistance genes could arise. Consequently, various novel alternatives to antimicrobials have been investigated for the control of dental plaque biofilms [8,13]. Notable examples include inhibitors of cell-cell signaling [14], enzyme-based technologies [15,16], and the use of oral probiotic organisms [17]. In addition, attention has focused upon the use of amino acids such as L-arginine that have been indicated to help prevent the development of cariogenic dental plaque biofilms [18,19,20,21]. Research into the mechanism of action of L-arginine has primarily centered on the ability of oral streptococci to catabolize L-arginine and consequently generate a local pH rise that counteracts the deleterious effects of acid on teeth [22,23]. Evidence also suggests that, while micromolar concentrations of L-arginine metabolically stabilize bacteria within coaggregates [24] and can mediate cell-cell signaling in dental plaque biofilms [24,25], millimolar concentrations can disaggregate bacterial coaggregates [26,27] and can influence the adhesion of *Streptococcus mutans* to tooth surfaces [21]. With a clear potential for a multifaceted role for L-arginine in biofilm metabolism and development, we hypothesized that L-arginine might *destabilize* oral multi-species biofilm communities. Specifically, destabilization would be expressed as changes in biofilm biomass, biofilm architecture and biofilm species community composition as a result of cell loss, altered cellular metabolism, and other effects caused directly or indirectly by L-arginine. Furthermore, because biofilms are inherently tolerant of antimicrobials, in-part due to slowed growth and restriction of access/penetration of antimicrobials, destabilized communities also might be more susceptible to antimicrobial treatments.

When studying oral biofilms, there has been considerable attention focused on *in vitro* laboratory model biofilm systems to develop biofilms of single-species or defined multi-species community composition that use artificial media [28,29,30,31]. While providing potentially useful baseline data for studies of biofilm developmental processes or the impact of candidate anti-biofilm compounds, single-species models ignore inter-species interactions that are a cornerstone for oral biofilm development [2]. Furthermore, the use of artificial media can generate biofilm communities that contain cells which are metabolically and phenotypically dissimilar to their normal state in flowing saliva [32,33]. Following recent technological advances, we have developed a flowing saliva microfluidic-based system that uses a Bioflux platform to evaluate the effect of anti-biofilm and antimicrobial compounds on multi-species biofilms grown under environmentally relevant conditions [34]. Specifically, the system does not require the use of defined species or artificial media at any stage of model preparation or use. Unlike the larger-scale systems such as constant depth film fermentors (CDFFs) and flowcells, ours only requires milliliters of filtered, pooled human saliva per experiment, as a media source and as little as 100 microliter inoculums of pooled human saliva. Furthermore, the Bioflux system is compatible with confocal laser scanning microscopy and technologies that facilitate the detection/quantification of microorganisms using culture-independent techniques. As such, the system offers an attractive *in vitro* model to evaluate the impact of L-arginine on multi-species biofilms that contain species often documented to exist in *in vivo* oral biofilms and grown under flowing conditions similar to those observed in the human oral cavity (saliva, pH, temperature, shear/flow).

This study aimed to employ two different model biofilm systems to examine the effects of L-arginine on multi-species oral biofilms: a technically simple static system and the controlled-flow Bioflux microfluidic system. Since L-arginine is inherently basic, and can exert confounding pH effects on biofilms, we focused on using L-arginine monohydrochloride (LAHCl) which displays a more neutral pH in saliva than L-arginine free base and has been demonstrated to be physiologically compatible for oral administration [18,35]. The static model biofilm system provided a high-throughput approach to optimize a bolus delivery of LAHCl and examine any gross anti-biofilm and antimicrobial effects while the controlled-flow system (the Bioflux model) allowed us to examine the long-term and short-term effects of LAHCl by providing continual flow and replenishment of nutrients which more closely mimicked the oral cavity. Both model systems allowed for the evaluation of the effects of LAHCl on oral multi-species biofilms developed from an inoculum of pooled cell-containing saliva with filter sterilized 25% pooled saliva used as the nutrient medium. Furthermore, both systems utilized confocal laser scanning microscopy to evaluate biofilm architecture and viability. Biofilms grown within the Bioflux system were subject to community composition analysis by 454 pyrosequencing. Here, we show that high millimolar concentrations of LAHCl alter biofilm three-dimensional architecture and biovolume in static saliva-based (microplate) and controlled-flow saliva-based (Bioflux) model systems, and we demonstrate that LAHCl can significantly alter multi-species biofilm community composition under natural flowing saliva conditions. In addition, we show that LAHCl can enhance the antimicrobial efficacy of cetylpyridinium chloride (CPC), a cationic antimicrobial that is commonly used in oral healthcare products but is subject to retarded biofilm penetration [34,36]. Collectively, findings from this work provide a framework for future fundamental and applied biofilm-control studies based on the use of LAHCl.

## Materials and Methods

### Nutrient and Inoculum Collection and Preparation

A saliva collection protocol, that did not collect the identifiers of individuals and facilitated the generation of pooled human saliva, was reviewed by the University of Michigan Health Sciences and Behavioral Sciences Institutional Review Board and given a “not regulated status” (Study eResearch ID: HUM00042954). As described by Nance and colleagues [34], saliva was collected from 6 individuals and pooled. The pooled saliva was used as either a cell-containing saliva (CCS) inoculum or as a cell-free saliva (CFS) nutrient source. CCS was prepared by mixing pooled saliva with glycerol (25% final glycerol concentration) before storing at -80°C. CFS was prepared by adding 2.5 mM of the reducing agent dithiothreitol (DTT) to pooled saliva followed by centrifugation at 17,000 x g for 30 min. The supernatant was mixed with sterile distilled water to make 25% saliva before filter-sterilizing through a 0.22 μm pore-size PES filter (Nalge Nunc International, Rochester, NY) and storage at -80°C.

### Static Microplate System

Static oral multi-species biofilms were developed in 24-well glass-bottom Sensoplate microplates (Greiner Bio-One, Monroe, NC) using CFS as the sole nutrient source and CCS as the inoculum. When required, L-arginine HCl (LAHCl) was added to CFS at final concentrations between 50 μM–500 mM (10-fold increments). 1.5 mL of CFS (negative control) and supplemented CFS of different LAHCl concentrations were added to each well. Wells were inoculated with 15 μL of CCS. After incubation at 37°C for 22 h, wells were treated with BacLight LIVE/DEAD viability stain (3.34 μM Syto 9 and 20 μM propidium iodide) (Invitrogen, Carlsbad, CA) according to the manufacturer’s instructions. After 30 min, the stained biofilms were washed with 1 mL of phosphate-buffered saline (PBS; pH: 7.4) three times, and examined microscopically.

### Flow-Based Microfluidic System

Using the approach of Nance and coworkers [34], 24-channel Bioflux plates (Fluxion, San Francisco, CA) were first treated with CFS to improve cell adhesion by flowing 100 μL of CFS across the inner-channel surfaces at 1 dyn/cm^2^ for 2 min and subsequently allowed to stand for 20 min. To inoculate the system, 100 μL of CCS were added to each outlet-well and flowed toward the inlet-well at 1 dyn/cm^2^ for 6 s. The plate was incubated at 37°C for 40 min to facilitate cell adhesion. Next, 900 μL of CFS (supplemented with or without LAHCl, described below) were added to each inlet-well and flowed toward the outlet well at 0.2 dyn/cm^2^ at 37°C for 20 h.

### Effect of Sustained Exposure of Different Concentrations of L-Arginine HCl (LAHCl) on Oral Biofilm Architecture and Viability

LAHCl was added to CFS to final concentrations between 500 μM and 500 mM. The supplemented or non-supplemented CFS was used as the medium source for the inoculated Bioflux flowing biofilm system and was added 40 min after initial inoculation with CCS. After 20 h incubation (37°C), the pH of the spent supplemented and non-supplemented CFS was checked and the developed biofilms were washed in PBS (pH 7.4; 20 min, 0.2 dyn/cm^2^) and stained using BacLight LIVE/DEAD viability stain according to the manufacturer’s instructions (Invitrogen, Carlsbad, CA) for 45 min at room temperature. Excess stain was removed by flowing 100 μL of PBS (pH 7.4) at 0.2 dyn/cm^2^ over biofilms for 20 min at room temperature.

### Culture-Independent Analysis of Oral Biofilm Community Composition

Biofilm cells were harvested and the samples were analyzed using bacterial tag encoded FLX amplicon pyrosequencing (bTEFAP), using the primers 939F (5’-TTGACGGGGGCCCGCAC-3’) and 1492R (5’-TACCTTGTTACGACTT-3’) by a method similar to that described by Nance and coworkers [34]. Briefly, cells from developed biofilms were harvested by pulsing sterile distilled water through the microfluidic channels at 20.0 dyn/cm^2^ (flow rate 745 mL/h, corresponding to a shear of 800 s^-1^) in both the forward and the reverse directions, in order to create shear stress to remove biofilm cells. This washing procedure was repeated until 90% or more (based upon microscopic inspection of surface-coverage) of the bound biofilm cells had been removed.

DNA was extracted from the harvested biofilm cells using a Qiagen DNA preparation system (Qiagen, Valencia, CA), according to the manufacturer’s instructions. Single step PCR was performed to generate barcoded amplicons with linkers. The concentration and size of DNA fragments were determined using DNA chips within a Bio-Rad Experion Automated Electrophoresis Station (Bio-Rad Laboratories, Hercules, CA) and a TBS-380 Fluorometer (Promega Corporation, Madison, WI). The 454 sequencing run was performed on a 70 × 75 GS PicoTiterPlate using a Genome Sequencer FLX System (Roche, Nutley, NJ). Sequence quality control was performed as described previously [37]. To determine the predicted identity of bacteria, remaining sequences were de-noised and de-replicated. OTU clustering was performed using uClust (http://www.drive5.com) and then queried using BLASTn against a custom highly curated database of 16S ribosomal RNA sequences derived from the National Center Biotechnology Information (http://www.ncbi.nlm.nih.gov/). BLASTn outputs were compiled and data reduction analysis was carried-out using the approach of Callaway et al. [38].

### Evaluation of the Ability of LAHCl to Augment the Activity of Antimicrobials

Using a 24-channel Bioflux plate and following 20 h growth in CFS (described above), wells were aspirated and biofilms were washed with PBS (pH 7.4). Biofilms were then exposed to antimicrobial/LAHCl mixtures for 60 s by flowing 100 μL of the mixtures over the biofilms at 2 dynes/cm^2^. Treatments included 0.05% cetylpyridinium chloride (CPC), 0.01% CPC, 0.05% CPC plus 500 mM LAHCl, 0.01% CPC plus 500 mM LAHCl, 500 mM LAHCl, or sterile distilled water (negative control). Wells were immediately aspirated before adding PBS (pH 7.4) to the inlet wells and flowed towards the outlet wells at 0.2 dynes/cm^2^ for 20 minutes to eliminate the residual treatment solutions. Biofilms were stained using the BacLight LIVE/DEAD stain, as described above.

### Microscopy, Computational Biofilm Rendering, and Image Analysis

Biofilms were examined using a Leica (Leica, Exon, PA) SPE confocal laser scanning microscope (CLSM) equipped with a 10X 0.4 NA HC PL APO infinity corrected objective lens (for static saliva biofilm microplate assays) or a 40X 1.25 NA HCX PL APO infinity corrected objective lens (for flowing saliva biofilm Bioflux assays). A 488 nm laser (15% power) was used to excite the LIVE/DEAD stained biofilms. For unbiased image acquisition and analyses, gain and offset settings were kept constant and the emission capture gates (Syto 9: EM: 510–540 nm and propidium iodide: EM: 620–650 nm) were set to be the same for every experiment. Imaris software (Bitplane, Zurich, Switzerland) was used to render and visualize biofilms. COMSTAT [39] and IMAGEJ [40] were used to assess parameters such as biofilm biovolume (the total volume occupied by fluorescent cells per unit of substratum area), average biofilm thickness (the mean biofilm thickness in a field of view), (dimensionless) biofilm roughness (a measure of how much the thickness of the biofilm varies), and viability (percentage of fluorescent green staining of biofilm by Syto 9). For image analysis using COMSTAT, all image stacks underwent manual thresholding checks that were based upon visual inspection of 3D biofilm renderings in IMARIS and histograms calculated in IMAGEJ.

### Statistical analyses

Two-tailed Student's T-tests that accounted for unequal variances were performed for all IMAGEJ and COMSTAT derived data. Values of p<0.05 were considered significant.

## Results

### Exposure to Millimolar Concentrations of L-Arginine HCl Alter Biofilm Architecture under Static Conditions

**Fig 1:**
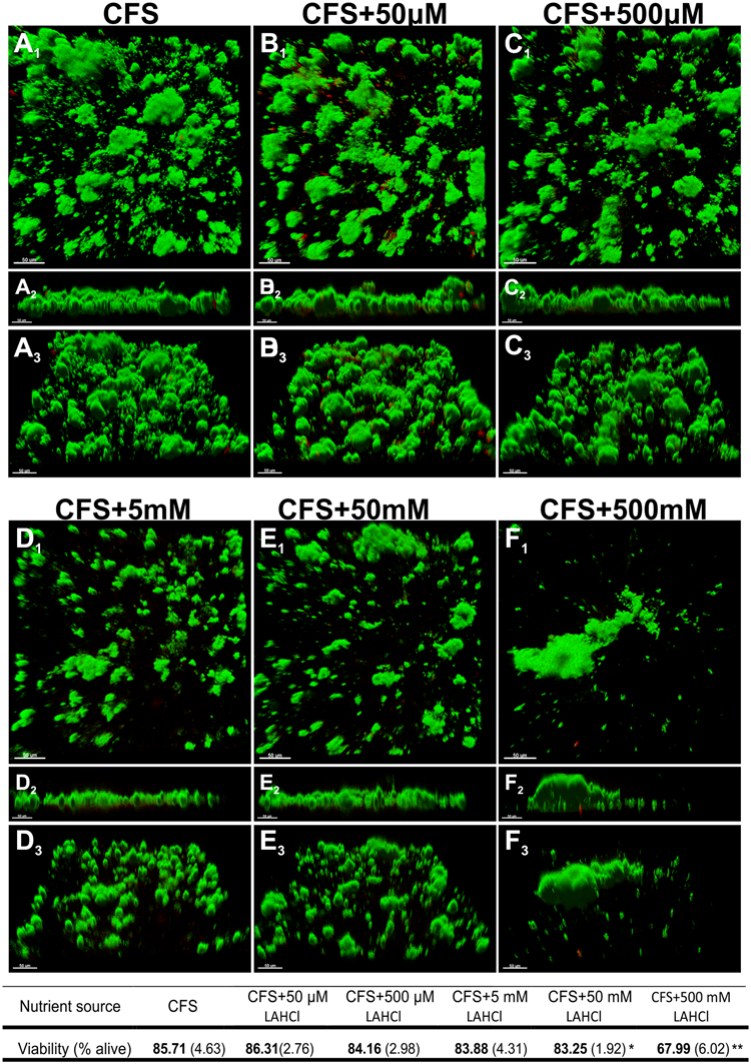
Differences in architecture of oral biofilms grown a static biofilm system containing different concentrations of L-arginine monohydrochloride (LAHCl). Images show representative 3D renderings of 22 h-old oral biofilms grown from a cell-containing saliva (CCS) inoculum in the static biofilm system containing cell free saliva (CFS) supplemented with different concentrations of LAHCl. Green signal (Syto 9) indicates viable cells and red signal (propidium iodide) indicates damaged/dead cells. Upper renderings (A_1_–F_1_) are of the x–y plane. Middle renderings (A_2_–F_2_) are of the x–z plane. Lower renderings (A_3_–F_3_) represent an angled view (x–y–z). Bars represent 50 μm. The associated table shows changes in percentage of cell viability with means presented in bold and standard deviations shown in parentheses (each derived from at least 27 images from nine biological replicates). *P<0.05; **P<0.01: significant differences from the CFS control.

Microcosm biofilms were developed in a static glass bottom microplate biofilm system that was inoculated with CCS and used CFS with increasing concentrations of LAHCl (50 μM–500 mM) as the growth medium. After 22 h growth, the architecture of the biofilms was observed to be substantially altered in a LAHCl concentration-dependent manner. Large biofilm towers that projected from surfaces into the overlying liquid were reduced at higher LAHCl concentrations in favor of smaller biofilm microcolonies with very occasional large biofilm masses (Fig 1A–1F). With the exception of 50 mM and 500 mM LAHCl, LIVE/DEAD staining of the biofilms grown at different LAHCl concentrations revealed that the supplementation of saliva with LAHCl consistently resulted in no statistically significant change in viability (Fig 1). Biofilms developed in 50 mM and 500 mM LAHCl displayed a modest but significant decrease in viability (Fig 1 associated table). However, visual inspection of the biofilms developed in 50 mM and 500 mM LAHCl suggested that the red signal was likely due to non-responsive/dead cells that are typically seen in multi-species biofilms but their proportion was increased, potentially due to the change in biofilm architecture and biomass (Fig 1E and 1F versus 1A–1D). Across all biofilm communities that were grown in CFS with or without LAHCl, red cells were always occasionally observed in small discrete patches that generally consisted of <10 cells of varying size and shape within or on the exterior regions of biofilms (Fig 1A–1F).

### Sustained Millimolar Concentrations of L-Arginine HCl Reduce Biofilms under Flowing Conditions

**Fig 2:**
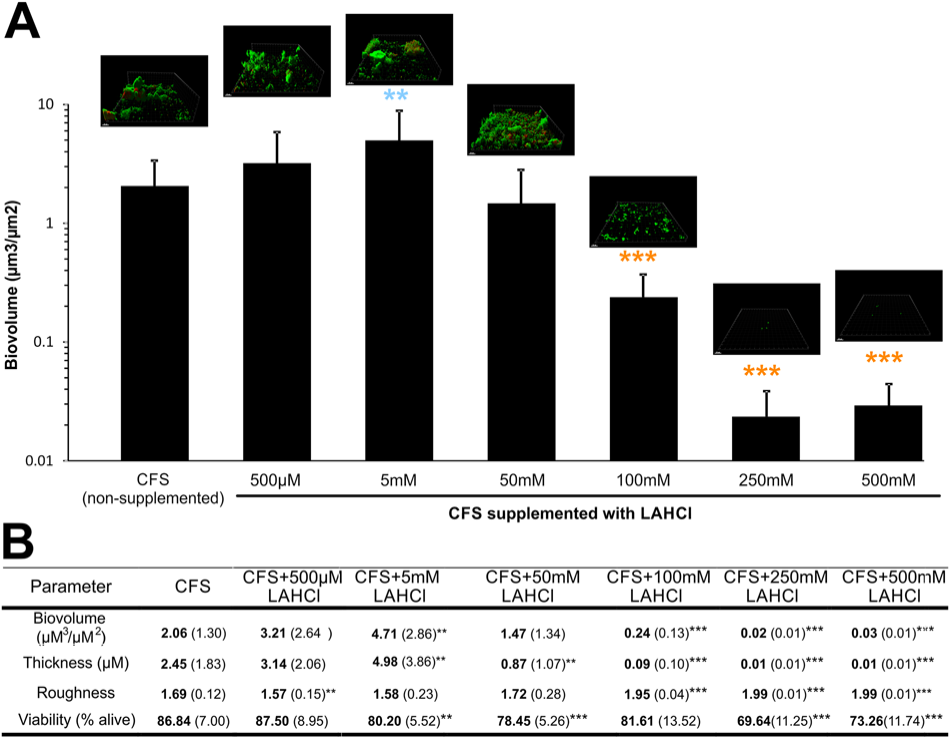
Effects of flowing saliva supplemented with different LAHCl concentrations on the development of oral biofilms for 20 h in the Bioflux system. The graph shows differences in biofilm biovolume and representative CLSM 3D renderings are included to highlight differences in biofilm architecture. Light blue colored star symbols embedded in the graph indicate a significant increase in average biofilm biovolume over CFS control and orange colored star symbols embedded in the graph indicate a significant decrease in average biofilm biovolume over CFS control. For the rendered biofilms within the embedded images, green colored cells indicate viable cells and red colored cells indicate damaged/dead cells. Bars represent 20 μm. The associated table shows, in addition to the biovolumes highlighted in the graph, changes in average biofilm thickness, average biofilm roughness, and cell viability. For data presented in the associated table, means are shown in bold and standard deviations are shown in parentheses (each derived from at least 18 images from six biological replicates). *P<0.05; **P<0.01; ***P<0.001: significant differences from the CFS control.

In the human oral cavity, saliva constantly flows over supragingival biofilms allowing for the mass-transfer of nutrients into the biofilm and the sloughing/de-adhesion/dispersion of biofilm cells to other areas. In addition, the flow of saliva maintains relatively stable concentrations of nutrients, in contrast to batch culture systems where nutrients such as arginine may be consumed by bacteria. To simulate the oral cavity more closely, a Bioflux microfluidic system was used to develop biofilms from inocula of CCS in flowing CFS supplemented with different concentrations of LAHCl. Microscopic inspection of LIVE/DEAD stained biofilms that were developed across the range of LAHCl concentrations revealed the presence of numerous morphologically distinct cell-types including cocci, rods, and fusiforms (S1 Fig). CLSM-derived renderings of the biofilms showed an inverse relationship between architectural complexity and exposure to increasing LAHCl. Specifically, as the LAHCl concentration increased, the ratio of small microcolonies to larger biofilm masses increased. By 100 mM LAHCl, no large biofilm masses were observed (CLSM renderings within Fig 2).

Quantitative image analysis using COMSTAT confirmed visual observations, and there were significant decreases in the biovolumes of biofilms developed in LAHCl concentrations of ≥100 mM (P<0.001, Fig 2). CFS supplemented with ≥100 mM LAHCl displayed a biovolume that was decreased by approximately 10–100 fold over the non-supplemented CFS control. A similar trend was also observed for average biofilm thickness. Roughness data supported the biovolume and thickness data (as well as the rendered images) suggesting that, when grown in higher millimolar LAHCl concentrations, the biofilms were much more sparse than the non-supplemented control. While an overall trend of a one-to-two order decrease in magnitude in biofilm biovolume was observed in the high millimolar range, at the low millimolar range, there was a concentration that supported a modest increase in biofilm biovolume. Biofilms developed within flowing CFS supplemented with an intermediate concentration (with respect to studied range) of 5 mM LAHCl displayed a two-fold increase in biovolume and thickness. The increase was accompanied by a large standard deviation and the biofilms contained the occasional large seemingly loosely-surface-attached microcolonies that flexed and moved in a gelatinous-like fashion as the microscope stage moved. There were even fewer occasional large loosely-attached microcolonies when biofilms were developed CFS supplemented with 50 mM LAHCl and above this concentration large biofilm masses were not observed.

Using IMAGEJ software, the percent viability of biofilms was derived from LIVE/DEAD stained biofilm cells. Viability measurements suggested that the destabilizing effects by LAHCl caused a small but significant decrease in viability at ≥5 mM LAHCl (except 100 mM LAHCl). Because of the seemingly indiscriminate location of dead/damaged biofilm cells and a broad-range of cell-types seen to be inactive, this was deemed to be possibly due to the random removal of viable cells from biofilm regions.

### Sustained Millimolar L-Arginine HCl Concentrations Alter Species Composition under Flowing Conditions

**Fig 3:**
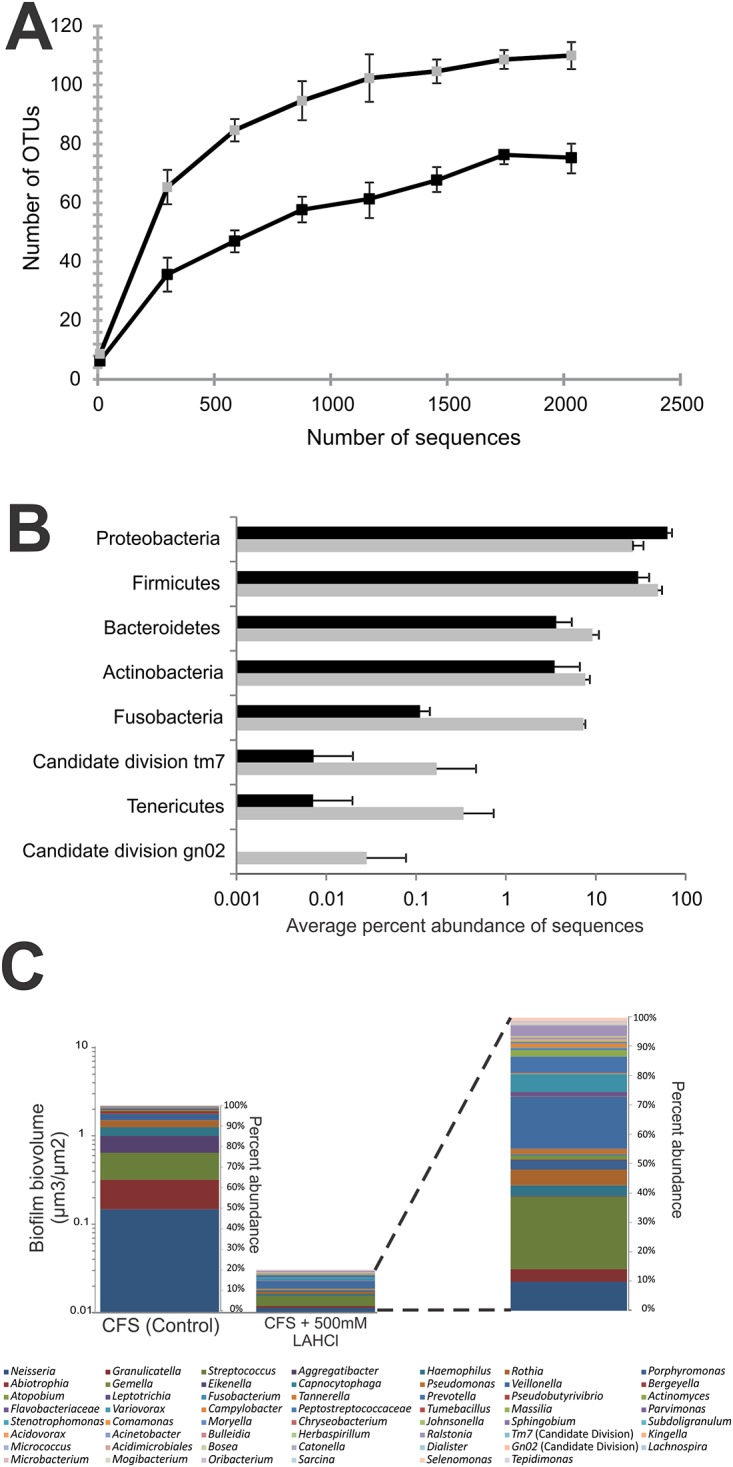
Sustained exposure to LAHCl alters biofilm community composition. Oral biofilms developed in the Bioflux system containing flowing CFS supplemented with 500 mM LAHCl display an altered biofilm community composition compared to those developed in non-supplemented CFS. Data derived from bacterial tag-encoded FLX amplicon pyrosequencing of oral biofilms grown for 20 h in three Bioflux microfluidic channels exposed to flowing CFS (control) and three Bioflux microfluidic channels exposed to flowing CFS supplemented with 500 mM LAHCl. (**A**) Comparison of OTU (97% identity) richness derived from rarefaction curves for biofilms developed in non-supplemented CFS (black square symbols) and biofilms developed in CFS supplemented with 500 mM LAHCl (grey square symbols). (**B**) Comparison of differences in abundance of phyla. Black bars represent data derived from the analysis of biofilms developed in flowing non-supplemented CFS while the grey bars represent data derived from the analysis of biofilms developed in CFS supplemented with 500 mM LAHCl. (**C**) Comparisons of differences in abundances of genera whereby abundances are normalized to biofilm biovolume (calculated from data presented in Fig 2). Note color coding (from left to right in the key) follows the order from most abundant in the CFS control. Far right color-coded bar is a magnified view of the community composition of the biofilm developed in CFS supplemented with 500 mM LAHCl. Supporting data is available in S1 Table.

Considering the striking difference in biofilm architecture that developed as a result of growth in flowing CFS supplemented with 500 mM LAHCl, we opted to use bTEFAP to compare community composition of the architecturally distinct biofilms to those formed in flowing non-supplemented CFS. Differences were observed in the total number of different genera, and the abundance of phyla/genera between biofilms developed in flowing non-supplemented CFS and 500 mM LAHCl supplemented CFS (Fig 3A, 3B, and 3C). Rarefaction analysis showed that even though biofilms grown in 500 mM LAHCl were reduced in biovolume (Fig 2), the diversity was almost two-fold greater than biofilms cultured in non-supplemented CFS (Fig 3A). At the phylum level, significant differences in the percent abundance of members of the Proteobacteria, Bacteroidetes, Fusobacteria, and Firmicutes were detected. The abundance of Proteobacteria in biofilms grown in flowing CFS supplemented with 500 mM LAHCl was less than half that in those grown in flowing non-supplemented CFS (Fig 3B). This decrease in abundance of Proteobacteria was mirrored by increase in abundances of Bacteroidetes, Fusobacteria, and Firmicutes within biofilms developed in flowing CFS supplemented with 500 mM LAHCl. The genera whose differences contributed to the decrease in abundance in the Proteobacteria phylum included *Neisseria* spp. (from an average of 49.76% to an average of 8.62%) and *Aggregatibacter* spp. (from an average of 8.16% to an average of 0.38%) (Fig 3C, S1 Table). The increase in abundances of the phyla Bacteroidetes, Fusobacteria, and Firmicutes was largely due to increases in *Prevotella* spp. (from an average 0.03% to an average 4.91%) *Fusobacterium* spp. (from an average 0.05% to an average 5.88%), *Veillonella* spp. (from an average 0.22% to an average 17.95%), and *Streptococcus* spp. (from an average 13.06% to an average 23.02%). From a total biomass perspective, it should be noted that changes in abundances of phyla/genera within the biofilms developed in flowing CFS supplemented with 500 mM LAHCl are also subject to the change in total *bioburden*: the absolute amount of biofilm cells/biofilm biomass, as inferred by CLSM and COMSTAT. Specifically, the development of biofilms in CFS supplemented with 500 mM LAHCl reduced biofilm biovolume by approximately 100-fold, when compared to non-supplemented saliva (highlighted in Fig 3C).

### Transient Exposure to L-Arginine HCl Reduces Biofilms and Enhances the Antimicrobial Activity of CPC

**Fig 4:**
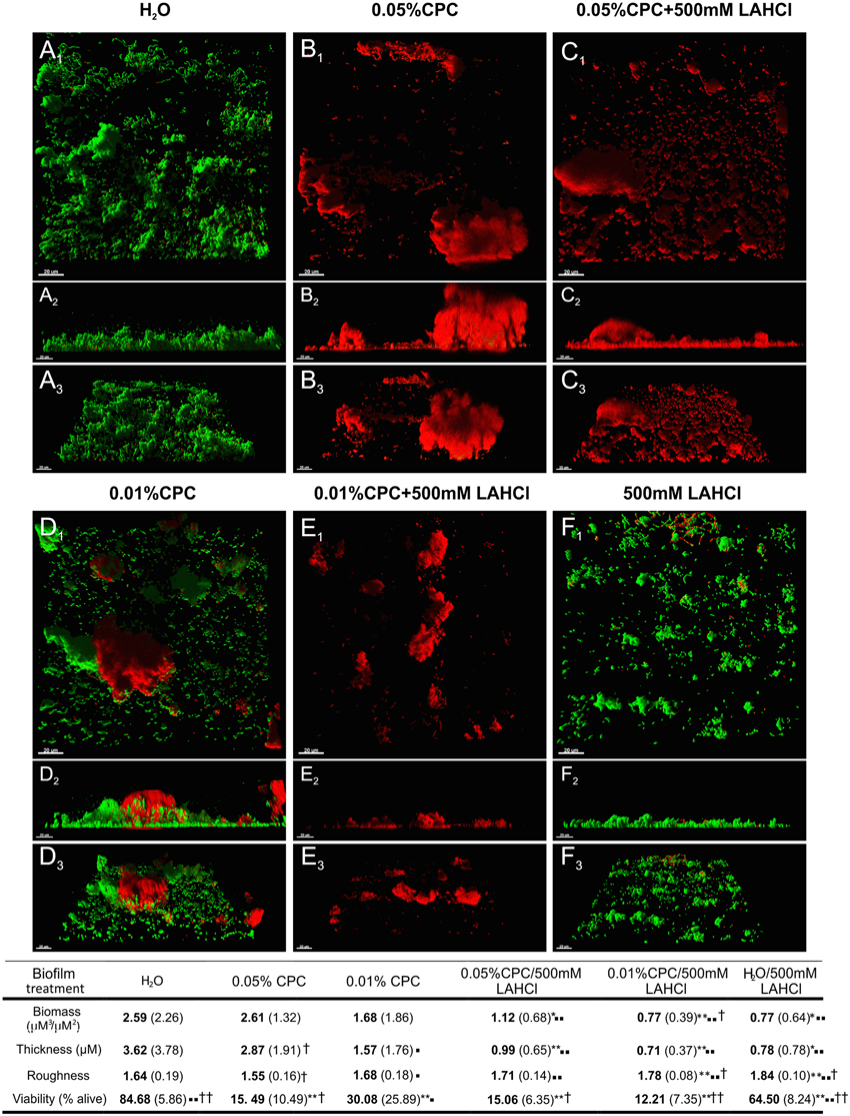
LAHCl affects preformed biofilms and enhances antimicrobial effectiveness. Demonstration that a 60 s exposure of oral multi-species biofilms developed in flowing cell free saliva (CFS) to solutions supplemented with LAHCl reduces biofilm biovolume and enhances antimicrobial efficacy of cetylpyridinium chloride (CPC). Representative biofilm renderings show 20 h oral biofilms developed from pooled CCS in flowing CFS in the Bioflux microfluidic system and subsequently exposed for 60 s to 0.05% or 0.01% CPC with or without 500 mM LAHCl. Green signal (Syto 9) shows viable cells, red signal (propidium iodide) shows damaged/dead cells. Upper images (A_1_–F_1_) are of the x–y plane. Middle images (A_2_–F_2_) are of the x–z plane. Lower images (A_3_–F_3_) are an angled view of each plane (x–y–z). Bars represent 20 μm. Associated table shows changes in cell viability, biofilm biovolume, thickness, and roughness. For data presented in the associated table, means are presented in bold and standard deviations are shown in parentheses (each derived from at least 9 images from three biological replicates). *P<0.05, **P<0.01: significant differences from the H_2_O control; ▪P<0.05, ▪▪P<0.01: significant differences from 0.05% CPC treatment; †P<0.05, ††P<0.01: significant differences from 0.01% CPC treatment.

Given that LAHCl destabilizes biofilm architecture, we tested whether high concentrations of LAHCl could enhance antimicrobial penetration and killing. Biofilms were developed from CCS in flowing non-LAHCl supplemented CFS for 20 h and then exposed for 60 s to 0.05% or 0.01% CPC either with or without 500 mM LAHCl (Fig 4). Three-dimensional rendering and image analysis demonstrated that treating oral biofilms with 500 mM LAHCl for 60 s reduced biofilm biovolume and thickness (P<0.05) as well as viability (P<0.01), presumably by causing de-adhesion of biofilm/coaggregated cells (Fig 4A versus 4F and associated table). Mixing 500 mM LAHCl with 0.05% CPC did not show enhanced killing/damage of biofilm cells, as compared with the non-LAHCl-augmented 0.05% CPC treatment (Fig 4B versus 4C and associated table), presumably because CPC was in excess. In contrast, mixing 500 mM LAHCl with a decreased amount of CPC (0.01%) resulted in significantly more (P<0.01) killing compared to the 0.01% CPC treatment (Fig 4D versus 4E and associated table). Three-dimensional computational rendering clearly supported image analysis data (Fig 4) and two data sets that were particularly contrasting was the 0.01% CPC treatment versus the 0.01% CPC treatment that was supplemented with 500 mM LAHCl. Rendering of the 0.01% CPC treated biofilm showed that killing was restricted to the upper parts of the biofilms (Fig 4D) while 0.01% CPC supplemented with 500 mM LAHCl resulted in almost complete penetration and killing (Fig 4E).

## Discussion

Using a static multi-species model biofilm system and a controlled-flow microfluidic multi-species model biofilm system, we demonstrated that L-arginine monohydrochloride (LAHCl) does not kill or damage biofilm bacteria but instead, appears to destabilize the biofilm in a concentration-dependent manner. Destabilization was inferred from the observed changes to architecture and biovolume occupied as well as relative proportions of the contained species. It is possible that LAHCl inhibited bacterial growth to some extent. However, by visualizing outlet wells from the Bioflux, there was evidence of growth even in the presence of 500 mM LAHCl (S2 Fig). Furthermore, the supplementation of 500 mM LAHCI with a cationic antimicrobial (CPC), which is common to many commercial mouthwashes, resulted in enhanced biofilm penetration and killing of cells. To our knowledge, this is the first demonstration that LAHCl can have multifaceted effects leading to changes in the community composition, architecture, and antimicrobial susceptibility of oral multi-species biofilms.

**Fig 5:**
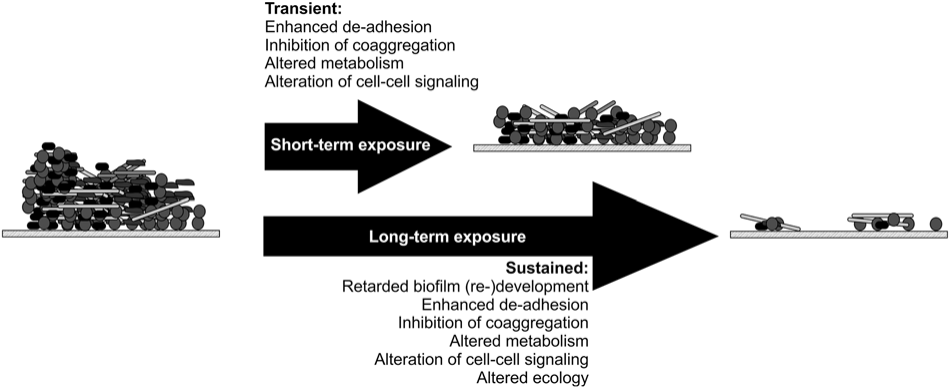
A model showing the proposed biofilm destabilizing effects of short-term exposure (transient; minutes) and longer-term exposure (sustained; hours) to high millimolar (≥100mM) LAHCl concentrations in flowing saliva. The model shows the effect of LAHCl on a well-developed multi-species biofilm (with respect to architecture and species composition) although similar destabilizing effects would occur on multi-species biofilms of younger or older developmental age. Cell shapes and sizes are not to scale. Postulated effects are based on data presented in this manuscript and on previous observations of the effects of L-arginine on bacterial cells and cell-cell interactions, as discussed in the body of the text.

Many approaches to control oral biofilms rely upon the use of chemical agents to exert direct bactericidal or bacteriostatic effects. These approaches have garnered concern as they will conceivably promote the development of antimicrobial resistance [10]. The use of LAHCl to instead destabilize multi-species oral biofilms, resulting in the reduction of biofilm bioburden, represents a potentially desirable alternative to antimicrobial treatments because the dispersed bacteria will be either swallowed or expelled during cleaning regimens. It is not clear if biofilm bacteria could become resistant to the effects of L-arginine (in this case in the form of the salt LAHCl) because the mechanism(s) of action of LAHCl has yet to be determined. We hypothesize that LAHCl has multifaceted effects on the stability of oral biofilms (Fig 5). These include a decrease in viscosity of extracellular polymeric substances produced by bacteria [41], an alteration in cellular metabolism that results in biofilm dispersion and reduced antibiotic tolerance [42], an imbalance in cell-cell metabolic communication [43], changes in local pH due to the break-down of L-arginine [44], alteration in cell-cell signaling [25], and disruption of coaggregation that conceivably results in cell-cell rearrangement in biofilms [45]. When considering the possible anti-coaggregation effects, it should be noted that it is not always entirely clear from the previous published studies of the effect of L-arginine on coaggregation whether the pH neutral salt (LAHCl) was used or if the more alkaline L-arginine free base was used [46,47,48,49]. Thus, pH might have had a role in inhibiting coaggregation in those previous studies. However, we have performed our own studies using both forms of L-arginine and both have been shown to inhibit coaggregation (S3 Fig). It is also possible that high concentrations of LAHCl may exert an osmotic pressure that causes loss of membrane integrity resulting in cell damage and/or death [50]. However, as inferred by LIVE/DEAD staining, cell death in the static or flowing biofilm systems was minimal and certainly much less than those levels documented for saliva-grown oral biofilms treated with antimicrobials [34] (Figs 1, 2, and S2 Fig). Collectively, the data suggest that the treatment of oral biofilms with LAHCl results in very limited cell-death, possibly due to the development of unfavorable biofilm conditions (e.g. changes in oxygen tension due to architectural changes to the biofilms); however, LAHCl does not exert direct antimicrobial effects. It is likely that the relative contribution of each of these LAHCl-induced effects depends upon not only the concentration of LAHCl but also duration of exposure (Fig 5). For example, short-term transient exposures that reduce biofilm biomass would likely be due to rapid de-adhesion events caused by inhibition of coaggregation (as well as inhibition of non-specific cell-cell adhesion). Long-term sustained exposure would likely have a multitude of effects including inhibition of coaggregation, altered metabolism by cells (caused directly by available L-arginine and indirectly by changes in the position of interacting species in the multi-species biofilm), and altered cell-cell signaling. An interesting study to follow-up the work presented here would be to examine how repeated short-term transient exposures affect the community over the long-term (Fig 5). This repeated dosing effect is especially relevant when one considers that this is the type of repetitive exposure that one would envisage for the treatment of natural biofilm communities within the human oral cavity.

Cationic antimicrobials are known to be less effective against slow-growing bacteria [51] and subject to reaction diffusion limitation, which limits penetration to deeper lying cells [4]. Considering that our data shows that LAHCl enhances the antimicrobial activity of CPC (Fig 4), it is likely that LAHCl enhances the metabolic activity of cells, making them more susceptible to CPC, and also allows for greater and more rapid biofilm penetration of CPC because of inhibition of coaggregation. It is also possible that LAHCl alters the sticky extracellular polysaccharide framework that aids in biofilm support/retention, which would also enhance CPC penetration [41]. However, more work needs to be performed on less complex (single- or dual-species) biofilm populations to fully elucidate how LAHCl enhances killing.

The most effective LAHCl concentrations for destabilizing biofilms were between 50 and 500 mM; lower concentrations displayed reduced effects. Formulations containing similar high millimolar concentrations of L-arginine have been commercialized for use in oral rinses and oral pastes to treat dentin hypersensitivity [52]. The mechanism of action of these L-arginine containing rinses and pastes centers upon the loading and occlusion of dentin tubules as well as the coating of tooth surfaces [53]. As such, a residual high concentration of L-arginine could remain in the oral cavity after initial exposure—resulting in sustained retention-times promoting long-term oral biofilm destabilization. Recently, clinical work has begun to look at the potential of L-arginine to modulate oral biofilm communities, by stimulating the production of alkali in the form of ammonia by bacterial arginine dihydrolase pathway enzymes [18,23]. This is hypothesized to contribute to changes in community composition in carious multi-species biofilms to select against cariogenic *S*. *mutans*. In this context, it would be interesting to examine the effect of L-arginine in the form of LAHCl on *in vitro* model multi-species biofilms containing *S*. *mutans* as well as revisiting the effect of LAHCl on *in vivo* biofilms as part of a clinical study.

An advantage to using the Bioflux microfluidic biofilm system, over other model biofilm systems, is that it allows us to replicate conditions found in the human oral cavity to generate multi-species biofilm communities that display a species composition similar to dental plaque [54]. Indeed, given the dominance of *Neisseria* species, the high levels of *Streptococcus* and *Granulicatella* species, and the numerous but less abundant characteristic anaerobic species (including members of the candidate division TM7 at low abundances), it is clear that the biofilms grown in the Bioflux microfluidic biofilm system contained species with abundances that are similar to that detected in human immature oral supragingival plaque [55,56,57]. Supplementing the saliva with 500 mM LAHCl not only altered biofilm architecture and reduced biofilm biovolume, but also changed the biofilm community from one that contained a large proportion of *Neisseria* spp. followed by *Granulicatella* and *Streptococcus* spp. to one that was dominated by *Streptococcus* spp. followed by *Veillonella*, *Neisseria* spp., and *Fusobacterium* spp. In particular, *Streptococcus* spp. are well-known to catabolize arginine [44] and it is possible that members of this genera benefitted directly from using L-arginine (in the form of LAHCl) as a nutrient. Further, *Veillonella* spp. often co-localize with streptococci since they use lactate, which is an end-product of streptococcal carbohydrate metabolism [58]. Thus, the increase in abundance of *Veillonella* spp. is potentially a consequence of the larger lactate-producing population of streptococci. The abundance of *Fusobacterium* spp. also increased in LAHCl treated biofilms (again, with respect to proportion of entire community). While an obligate anaerobe and typically only detected in more mature biofilm communities, the increase in abundance of *Fusobacterium* spp. may partly be accounted for by the ability of this organism to coaggregate with many oral species. Some coaggregation interactions may be inhibited by the presence of LAHCl [59,60] but other coaggregation interactions may not [61]. Thus, the *multigeneric* coaggregation-mediated *bridging ability* of *Fusobacterium* spp. [5,62] may give it an ability to reside in LAHCl containing environments when other species are removed. In context with a very recent clinical study, L-arginine was also shown to affect the composition of dental plaque in caries-free and caries-active subjects, as measured by microarray [18]. However, it is difficult to compare the data with our observations due to the very different *in vivo* and *in vitro* techniques employed and different research focus. Nevertheless, it is interesting to note that in that clinical study the proportions of *Neisseria*, *Fusobacterium* and *Veillonella* were also altered by L-arginine.

Given the destabilizing effects of high millimolar concentrations of LAHCl, as well as the increasing demand to develop more efficacious oral healthcare technologies, the prospect of using L-arginine salts such as LAHCl to improve oral health needs to be rigorously assessed. As well as fundamental mechanistic studies, clinical investigations exploring the use of L-arginine salts in antimicrobial formulations would also be potentially fruitful. Indeed, it is also possible that the treatment of biofilms with LAHCl may extend beyond oral applications and may be of use in destabilizing biofilms in other environments such as in wounds, catheters, and other medically or environmentally relevant situations.

## Supporting Information

S1 Fig Magnified and digitally enhanced three dimensional reconstructions showing examples of cell-types and cell arrangements within oral biofilms developed in the Bioflux microfluidic system. (**A**) A mixture of cell-types in coaggregated microcolonies in a biofilm developed in CFS. (**B**) A fusiform-like cell with coaggregated cocci that is partially exposed and projecting from microcolonies in a biofilm developed in CFS. (**C**) Long chains of streptococci in loose masses in a biofilm developed in CFS supplemented with 100 mM LAHCl. (**D**). A microcolony of densely-packed cocci in a biofilm developed in CFS supplemented with 500 mM LAHCl. Bars represent 5 μm.
(TIF)

S2 Fig Presence or absence of biofilm through the Bioflux microfluidic system following 20 h growth at 37°C in CFS or CFS containing 500 mM LAHCl. System was flooded with LIVE/DEAD stain to highlight which cells were alive (green) or damaged/dead (red). (**A**) When grown in CFS that was not supplemented with LAHCl, no substantial biofilm was observed in the inlet well (very occasional fluorescent material was detected) and sizeable biofilms were observed in the channels and outlet wells. (**B**) When grown in CFS supplemented with 500 mM LAHCl, no substantial biofilm was observed in the inlet well (very occasional fluorescent material was detected), no substantial biofilm was observed in the channels (mostly small microcolonies of cells), but large biofilm masses were seen in the outlet well. Note there was no visible reduction in viability in the presence of LAHCl in the outlet well. Blue circles represent area of evaluation for biofilm. Bars represent 30 μm.
(TIF)

S3 Fig Supplementing coaggregation buffer with either LAHCl or L-arginine (free base) inhibits coaggregation between three species of oral bacteria. Cells of the oral species *Streptococcus gordonii* DL1 (SgDL1), *Streptococcus oralis* 34 (So34), and *Actinomyces oris* T14V (AoT14V) were grown in batch culture and suspended in coaggregation buffer according to the method of Cisar and colleagues [63]. Suspensions of equal cell density (optical density of 1.5 at 600nm; **A-C**) were then mixed in equal volumes (400μl of each species) in coaggregation buffer (**D-F**) or coaggregation buffer supplemented with either LAHCl (**G-I**) or L-arginine (free base) (**J-L**). Visual scores ranging from 0 (no coaggregation) through 4+ (maximum coaggregation) were assigned using the criteria of Cisar and colleagues [63]. Scale bar represents 5 mm.
(TIF)

S1 Table Species composition of biofilms developed from pooled CCS inoculums in the Bioflux biofilm system containing flowing CFS supplemented with or without 500 mM LAHCl. Average percentage abundance of each species based on bacterial tag-encoded FLX amplicon pyrosequencing of oral biofilms grown for 20 h in three Bioflux microfluidic channels exposed to flowing CFS (control) and in three Bioflux microfluidic channels exposed to flowing CFS supplemented with 500 mM LAHCl. Unspeciated sequences are assigned to respective genera and highlighted in bold. Values are rounded to two decimal places. It should be noted that relative abundances are subject to primer bias and are therefore approximate.
(PDF)
