# Supplementary figures and images for: L-Arginine Destabilizes Oral Multi-Species Biofilm Communities Developed in Human Saliva
Source: PLoS One. 2015 May 6;10(5):e0121835. doi: 10.1371/journal.pone.0121835 (PMC4422691; doi:10.1371/journal.pone.0121835)

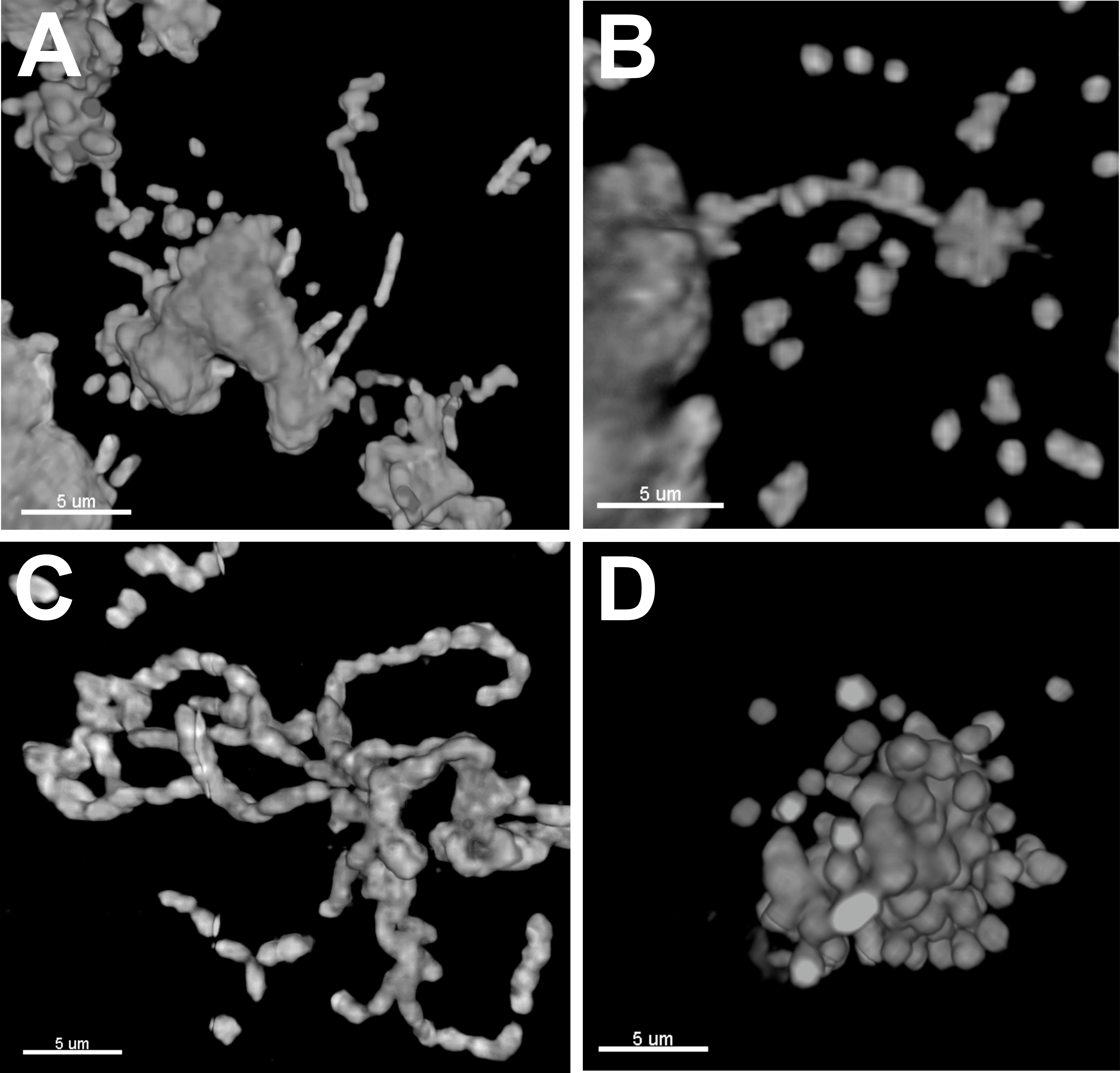

Supplement: S1 Fig — (A) A mixture of cell-types in coaggregated microcolonies in a biofilm developed in CFS. (B) A fusiform-like cell with coaggregated cocci that is partially exposed and projecting from microcolonies in a biofilm developed in CFS. (C) Long chains of streptococci in loose masses in a biofilm developed in CFS supplemented with 100 mM LAHCl. (D). A microcolony of densely-packed cocci in a biofilm developed in CFS supplemented with 500 mM LAHCl. Bars represent 5 μm. (TIF) [file pone.0121835.s001.tif]

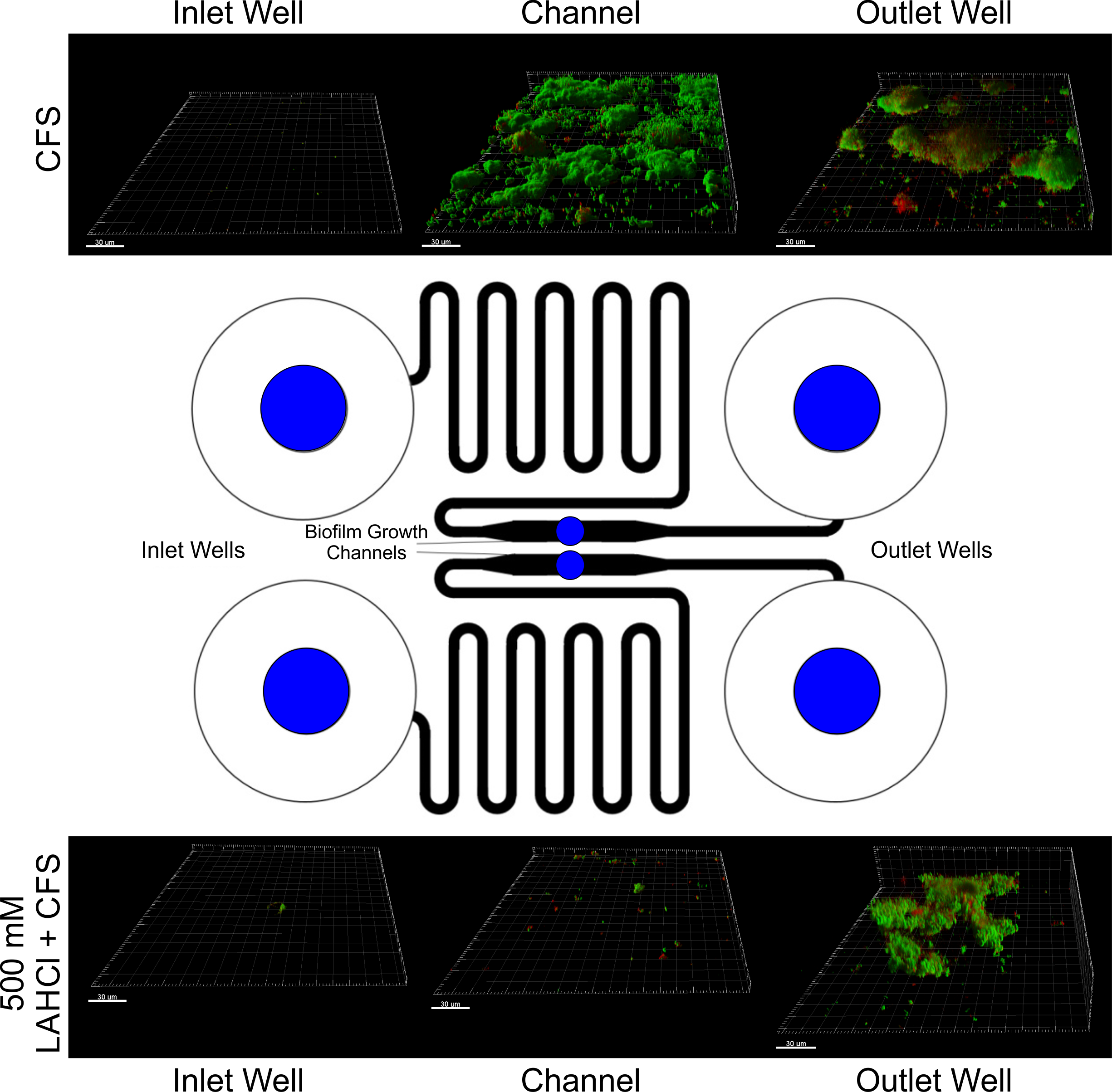

Supplement: S2 Fig — System was flooded with LIVE/DEAD stain to highlight which cells were alive (green) or damaged/dead (red). (A) When grown in CFS that was not supplemented with LAHCl, no substantial biofilm was observed in the inlet well (very occasional fluorescent material was detected) and sizeable biofilms were observed in the channels and outlet wells. (B) When grown in CFS supplemented with 500 mM LAHCl, no substantial biofilm was observed in the inlet well (very occasional fluorescent material was detected), no substantial biofilm was observed in the channels (mostly small microcolonies of cells), but large biofilm masses were seen in the outlet well. Note there was no visible reduction in viability in the presence of LAHCl in the outlet well. Blue circles represent area of evaluation for biofilm. Bars represent 30 μm. (TIF) [file pone.0121835.s002.tif]

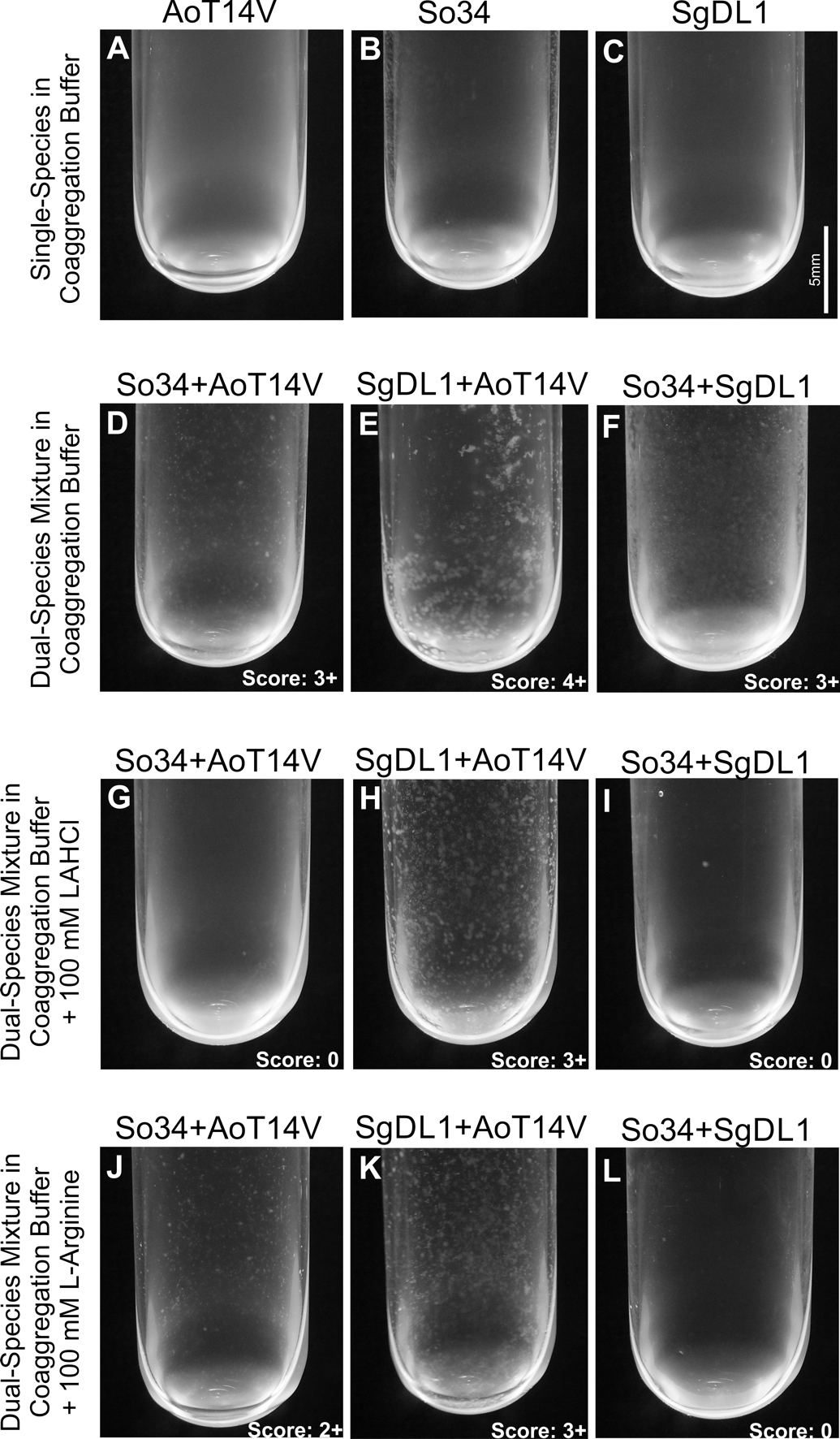

Supplement: S3 Fig — Cells of the oral species Streptococcus gordonii DL1 (SgDL1), Streptococcus oralis 34 (So34), and Actinomyces oris T14V (AoT14V) were grown in batch culture and suspended in coaggregation buffer according to the method of Cisar and colleagues [63]. Suspensions of equal cell density (optical density of 1.5 at 600nm; A-C) were then mixed in equal volumes (400μl of each species) in coaggregation buffer (D-F) or coaggregation buffer supplemented with either LAHCl (G-I) or L-arginine (free base) (J-L). Visual scores ranging from 0 (no coaggregation) through 4+ (maximum coaggregation) were assigned using the criteria of Cisar and colleagues [63]. Scale bar represents 5 mm. (TIF) [file pone.0121835.s003.tif]
