# Supplementary material for: L-Arginine Destabilizes Oral Multi-Species Biofilm Communities Developed in Human Saliva
Source: PLoS One. 2015 May 6;10(5):e0121835. doi: 10.1371/journal.pone.0121835 (PMC4422691; doi:10.1371/journal.pone.0121835)
Supplement: S1 Table — Average percentage abundance of each species based on bacterial tag-encoded FLX amplicon pyrosequencing of oral biofilms grown for 20 h in three Bioflux microfluidic channels exposed to flowing CFS (control) and in three Bioflux microfluidic channels exposed to flowing CFS supplemented with 500 mM LAHCl. Unspeciated sequences are assigned to respective genera and highlighted in bold. Values are rounded to two decimal places. It should be noted that relative abundances are subject to primer bias and are therefore approximate. (PDF) [file pone.0121835.s004.pdf]

S1 Table. Species composition of biofilms developed from pooled CCS inoculums in the Bioflux biofilm system containing flowing CFS supplemented with or without 500 mM LAHCl. Average percentage abundance of each species based on bacterial tag-encoded FLX amplicon pyrosequencing of oral biofilms grown for 20 h in three Bioflux microfluidic channels exposed to flowing CFS (control) and in three Bioflux microfluidic channels exposed to flowing CFS supplemented with 500 mM LAHCl. Unspeciated sequences are assigned to respective genera and highlighted in bold. Values are rounded to two decimal places. It should be noted that relative abundances are subject to primer bias and are therefore approximate.

Species	Average across 3 channels (CFS)	Standard deviation across 3 channels (CFS)	Average across 3 channels (500 mM LAHCl+CFS)	Standard deviation across 3 channels (500 mM LAHCl+CFS)
Abiotrophia defectiva	1.36	1.19	0.26	0.01
**Unspeciated *Acidimicrobiales***	0.00	0.00	0.08	0.08
**Unspeciated *Acidovorax***	0.01	0.01	0.53	0.38
Acinetobacter genomosp.3	0.00	0.00	0.02	0.03
Acinetobacter schindleri	0.01	0.01	0.33	0.09
Actinomyces graevenitzii	0.00	0.00	0.35	0.11
Actinomyces lingnae	0.00	0.00	0.24	0.19
Actinomyces naeslundii	0.00	0.00	0.06	0.05
Actinomyces odontolyticus	0.03	0.04	1.48	0.54
Actinomyces oris	0.00	0.00	0.02	0.03
Actinomyces viscosus	0.00	0.00	0.02	0.03
Aggregatibacter aphrophilus	0.00	0.00	0.05	0.05
Aggregatibacter segnis	8.16	7.07	0.33	0.24
Atopobium parvulum	0.06	0.10	0.02	0.03
**Unspeciated *Bergeyella***	0.09	0.07	0.20	0.13
Bosea vestrisii	0.00	0.00	0.05	0.08
Bulleidia solobacterium moorei	0.01	0.01	0.34	0.39
Campylobacter concisus	0.02	0.02	1.15	0.78
Campylobacter mucosalis	0.00	0.00	0.09	0.09
Campylobacter showae	0.00	0.01	0.23	0.07
Capnocytophaga gingivalis	0.04	0.01	0.01	0.02
Capnocytophaga leadbetteri	0.06	0.02	0.06	0.07
Capnocytophaga sputigena	0.15	0.14	0.18	0.16
**Unspeciated *Capnocytophaga***	0.08	0.02	0.03	0.04
Catonella morbi	0.00	0.00	0.12	0.07
Chryseobacterium stagni	0.01	0.02	0.00	0.00
Comamonas denitrificans	0.01	0.01	0.07	0.11
Dialister invisus	0.00	0.00	0.02	0.04
Eikenella corrodens	0.43	0.27	0.14	0.12
Escherichia coli	0.01	0.01	0.01	0.01
Escherichia coli O26:H11	0.00	0.01	0.64	0.79
Escherichia fergusonii	0.00	0.00	0.03	0.03
Eubacterium saburreum	0.01	0.02	0.24	0.18
**Unspeciated *Flavobacteriaceae***	0.03	0.06	0.43	0.42
Fusobacterium nucleatum	0.00	0.01	0.52	0.87
Fusobacterium periodonticum	0.05	0.04	5.36	1.06
Gemella haemolysans	0.05	0.04	0.39	0.36
Gemella morbillorum	0.16	0.03	0.19	0.05
Gemella sanguinis	0.40	0.39	1.05	0.80
GN02 (candidate division)	0.00	0.00	0.03	0.05
Granulicatella adiacens	9.45	3.04	1.72	0.45
Granulicatella elegans	4.38	3.36	2.19	0.93
Granulicatella paradiacens	0.18	0.01	0.05	0.02
Granulicatella spp.	0.37	0.12	0.10	0.17
Haemophilus haemolyticus	0.00	0.00	0.03	0.06
Haemophilus parainfluenzae	3.17	2.60	3.51	1.22
Haemophilus quentini	0.00	0.00	0.13	0.18
**Unspeciated *Haemophilus***	1.07	0.99	0.10	0.01
Herbaspirillum putei	0.01	0.01	0.60	0.52
Kingella oralis	0.00	0.01	0.14	0.13

**Unspeciated *Kluyvera***	0.00	0.00	0.01	0.02
**Unspeciated *Lachnospira***	0.00	0.00	0.08	0.09
Leptotrichia buccalis	0.00	0.00	0.11	0.16
**Unspeciated *Leptotrichia***	0.06	0.04	0.41	0.31
Leptotrichia trevisanii	0.00	0.00	0.91	0.11
**Unspeciated *Massilia***	0.02	0.02	0.00	0.00
**Unspeciated *Microbacterium***	0.00	0.00	0.30	0.12
Micrococcus luteus	0.00	0.01	0.00	0.00
Mogibacterium pumilum	0.00	0.00	0.16	0.14
**Unspeciated *Moryella***	0.01	0.02	0.01	0.02
Neisseria cinerea	0.00	0.00	0.39	0.36
Neisseria elongata	0.02	0.02	0.01	0.01
Neisseria flavescens	28.10	10.31	5.54	2.13
Neisseria meningitidis	4.22	1.01	0.84	0.41
Neisseria mucosa	0.14	0.15	0.00	0.00
Neisseria sicca	2.62	2.37	0.17	0.09
Neisseria subflava	6.63	2.31	1.11	0.55
**Unspeciated *Neisseria***	8.03	8.99	0.56	0.21
Oribacterium sinus	0.00	0.00	0.24	0.23
Parvimonas micra	0.02	0.02	0.09	0.08
Peptostreptococcaceae spp.	0.02	0.02	0.30	0.38
Porphyromonas endodontalis	0.00	0.00	0.04	0.04
**Unspeciated *Porphyromonas.***	3.09	1.79	2.83	0.60
Prevotella histicola	0.00	0.00	0.15	0.17
Prevotella melaninogenica	0.00	0.00	1.79	0.58
Prevotella nanceiensis	0.00	0.00	0.60	0.49
Prevotella pallens	0.01	0.01	0.47	0.20
Prevotella salivae	0.00	0.00	0.52	0.28
Prevotella shahii	0.01	0.01	0.67	0.61
**Unspeciated *Prevotella***	0.01	0.03	0.71	0.09
**Unspeciated *Pseudobutyrivibrio***	0.04	0.06	0.00	0.00
Pseudomonas fluorescens	0.27	0.34	2.46	1.89
Pseudomonas geniculata	0.02	0.02	0.10	0.15
Pseudomonas pseudoalcaligenes	0.00	0.01	0.02	0.04
Pseudomonas trivialis	0.00	0.01	0.00	0.00
Ralstonia pickettii	0.01	0.01	5.89	8.61
Rothia aeria	0.00	0.00	0.39	0.04
Rothia mucilaginosa	3.39	3.21	4.70	0.93
**Unspeciated *Sarcina***	0.00	0.00	0.02	0.03
Selenomonas noxia	0.00	0.00	0.04	0.05
Selenomonas sputigena	0.00	0.00	0.06	0.08
**Unspeciated *Selenomonas***	0.00	0.00	1.04	0.30
Shigella flexneri	0.00	0.01	0.16	0.13
Sphingobium yanoikuyae	0.01	0.02	0.01	0.02
Streptococcus australis	0.53	0.19	3.60	2.33
Streptococcus cristatus	0.00	0.00	0.14	0.15
Streptococcus genomosp. C3	0.03	0.02	0.00	0.00
Streptococcus genomosp. C7	0.00	0.00	0.08	0.11
Streptococcus gordonii	0.09	0.00	0.06	0.11
Streptococcus infantis	2.03	0.68	4.33	0.44
Streptococcus intermedius	0.00	0.00	0.20	0.09
Streptococcus mitis	1.16	0.35	1.45	0.28
Streptococcus oralis	6.25	3.20	5.84	1.50
Streptococcus parasanguinis	0.30	0.13	1.26	0.31
Streptococcus parasanguinis motherC3	0.32	0.26	2.42	0.61
Streptococcus pseudopneumoniae	0.09	0.08	0.18	0.07
Streptococcus salivarius	0.07	0.07	1.61	0.29
Streptococcus sanguinis	1.01	0.62	1.02	0.43
**Unspeciated *Streptococcus***	1.18	0.48	0.83	0.30
**Unspeciated *subdoligranulum***	0.01	0.02	0.00	0.00
**Unspeciated *Tannerella***	0.05	0.04	0.50	0.39
**Unspeciated *Tepidimonas***	0.00	0.00	0.02	0.04
TM7 (candidate division)	0.01	0.01	0.17	0.29
**Unspeciated *Tumebacillus***	0.02	0.04	0.00	0.00
**Unspeciated *Variovorax***	0.03	0.03	0.54	0.37
Veillonella dispar	0.05	0.01	2.83	0.64
Veillonella parvula	0.10	0.09	7.50	1.70
**Unspeciated *Veillonella.***	0.07	0.05	7.62	1.74
